# The Key Role of GM1 Ganglioside in Parkinson’s Disease

**DOI:** 10.3390/biom12020173

**Published:** 2022-01-21

**Authors:** Suman Chowdhury, Robert Ledeen

**Affiliations:** Department of Pharmacology, Physiology, and Neuroscience, Rutgers, The State University of New Jersey, Newark, NJ 07103, USA; sc2131@njms.rutgers.edu

**Keywords:** Parkinson’s disease, GM1, ganglioside, multi-system disorder, neuroprotection

## Abstract

We have endeavored in this review to summarize our findings, which point to a systemic deficiency of ganglioside GM1 in Parkinson’s disease (PD) tissues. These include neuronal tissues well known to be involved in PD, such as substantia nigra of the brain and those of the peripheral nervous system, such as the colon and heart. Moreover, we included skin and fibroblasts in the study as well as peripheral blood mononuclear cells; these are tissues not directly involved in neuronal signaling. We show similar findings for ganglioside GD1a, which is the metabolic precursor to GM1. We discuss the likely causes of these GM1 deficiencies and the resultant biochemical mechanisms underlying loss of neuronal viability and normal functioning. Strong support for this hypothesis is provided by a mouse PD model involving partial GM1 deficiency based on mono-allelic disruption of the B4galnt1 gene. We point out that progressive loss of GM1/GD1a occurs in the periphery as well as the brain, thus obviating the need to speculate PD symptom transfer between these tissues. Finally, we discuss how these findings point to a potential disease-altering therapy for PD:GM1 replacement, as is strongly implicated in animal studies and clinical trials.

## 1. Introduction

**Figure 1:**
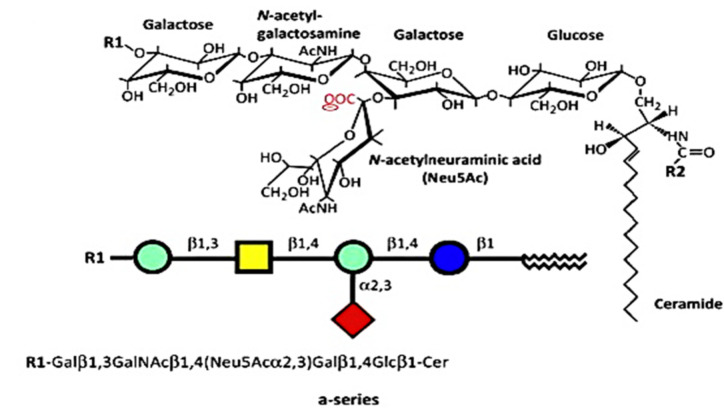
Structures of a-series. GM1 (GM1a), R1 = H, R2 = long-chain fatty acid (e.g., 18:0); GD1a, R1 = Neu5Acα2,3, R2 = long-chain fatty acid (e.g., 18:0); LIGA20, R1 = H, R2 = CHCl_2_ (image used are adapted from [4] with permission granted by Trends in biochemical sciences, Copyright 2015 Cellpress).

The numerous glycosphingolipids that occur in the nervous system and elsewhere are clearly involved in metabolic and pathological changes that accompany Parkinson’s disease [1], but one such molecule, GM1 ganglioside, has received focused attention from several workers for its prominent role in both the etiology and potential treatment of this neurodegenerative condition. GM1 is particularly abundant in neurons and is essential for their complex functioning (see below). The same may be said for ganglioside GD1a, which is identical in structure to GM1 but possesses an additional sialic acid attached to the terminal galactose of GM1 (Figure 1) [2]. That terminal sialic acid is readily removed by NEU3 neuraminidase, which is situated close to GD1a in the neuronal membrane [3]. This is why GD1a is considered to function as a reserve pool for GM1 as its primary function.

This review attempts to summarize our recently published evidence showing that the salient feature of GM1 in regard to PD is its subnormal level, especially in neurons but in other cell types as well. Such deficiency prevents the normal functioning of cells dependent on an adequate level of this ganglioside, resulting in their gradual quiescence and eventual death. The following section describes our studies documenting what appears to be a systemic deficiency of GM1 (and usually GD1a as well) in virtually all tissues of PD subjects. 

## 2. GM1 Deficiency Induces Parkinsonism in Mouse PD Model

One of the first clues suggesting that GM1 deficiency constitutes a major risk factor in PD came from the serendipitous observation of mice deficient in GM1 due to biallelic disruption of B4galnt1 (GM2 synthase-B4galnt1–/–) [5]. GM2 is the obligatory precursor to GM1. In addition to motor dysfunction, these mice showed several neuropathologies characteristic of PD, including the key feature of aggregated alpha-synuclein. Additional symptoms included the depletion of striatal dopamine (DA) and loss of DA neurons of the substantia nigra pars compacta (SNpc). Movement disorder was alleviated by L-dopa administration, and neuropathologies were alleviated by LIGA20, an analog of GM1 structurally similar to the latter (Figure 1) but is more membrane permeable and, hence, more active. GM1 itself was not effective in those initial studies due to inadequate dosages. However, other investigators showed GM1 to be therapeutically effective in PD mice under appropriate conditions [2; for review].

**Figure 2:**
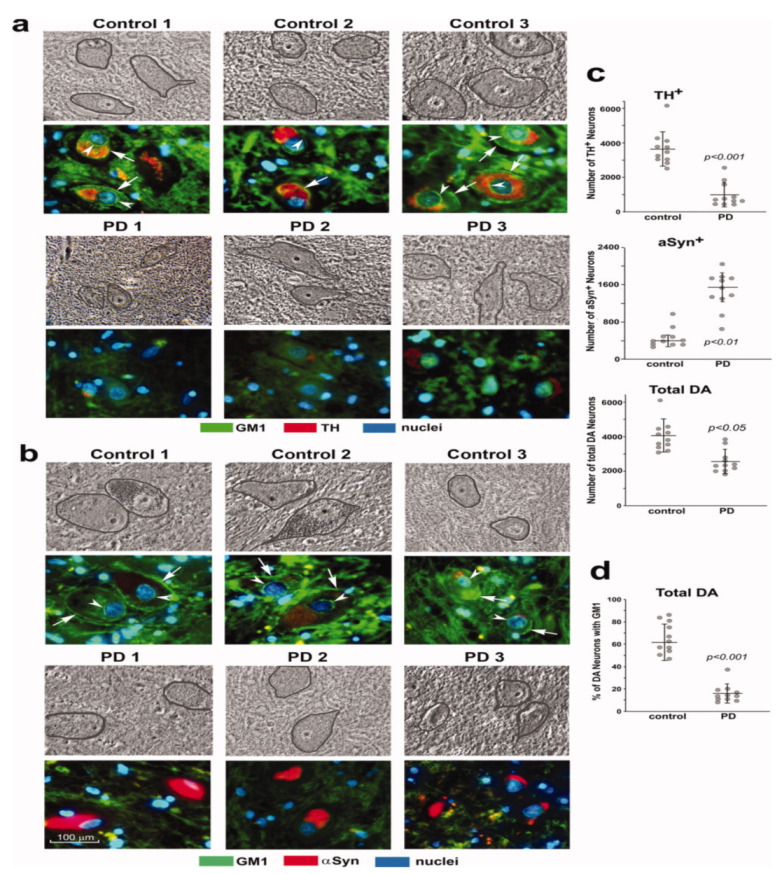
GM1 deficiency in SNpc neurons of PD patients. Immunohistochemical staining of GM1 and TH (**a**) and GM1 plus aSyn (**b**) in paraffin sections from SNpc region of three PD patients and three non-PD controls. Hoechst 33342-stained nuclei. DA neurons are encircled in phase images shown in upper panels. GM1 expression is indicated in both plasma membrane (arrows) and nuclear envelope (arrowheads). (**c**,**d**): Quantification for 11 sporadic PD patients and 11 non-PD controls. Eight sections of each patient were counted, with total DA neurons (TH^1^plus aSyn^1^) ranging from 3100 to 6500 in non-PD controls and from 1400 to 3800 in PD patients. Data are mean ± SD. Statistical difference was analyzed with two-tailed Student’s *t*-test (images used are adapted from [6] with permission granted by Journal of Neuroscience Research, Copyright 2012 Wiley Online Library).

Study [6] was particularly revealing, in which mice with only monoallelic disruption of the same gene (B4galnt1+/−) manifested Parkinsonian symptoms closely similar to those of the above biallelic mice; such mice had only partial deficiency of GM1. In order to test the possibility that the same might apply to PD itself, we carried out immunohistochemical analysis of paraffin sections from the SNpc of PD subjects [6]. These showed substantially less GM1 in nigral DA neurons than in age-matched controls (Figure 2). Soon after that, we came into the possession of tissues from the occipital cortex of PD patients, and we measured their GM1 by the well-established method of high performance thin-layer chromatography (HPTLC) [7]. This too showed a significant deficiency of GM1, even though that part of the brain is not intimately involved in PD pathology. These findings prompted us to examine additional tissues to observe how widespread GM1 deficiency actually is in PD.

## 3. Systemic Deficiency of GM1 in PD Tissues

**Figure 3:**
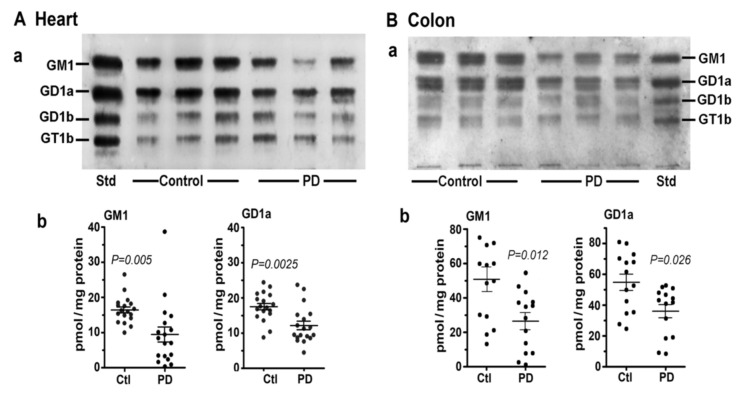
Gangliosides in heart and colon from PD patients and age-matched controls. (**A**): Heart (*n* = 18 in each group). (**B**): Colon (*n* = 14 in each group). In (**A**,**B**), subpanel (**a**) is HPTLC, and subpanel (**b**) is densitometry quantification showing the statistical difference between PD and controls, determined by Mann–Whitney rank sum U test (images used are adapted from [8] with permission granted by Glycoconjugate J., Copyright 2021 Springer).

In this study [8] we first examined two tissues that are well recognized as major participants in PD pathology: colon and heart. Multiple samples of these along with age-matched controls were obtained from the Banner Sun Health Research Inst. (Sun City, AZ, USA; Dr Thomas Beach). Total lipids were extracted with chloroform (C): methanol (M) (1:1 by vol), and after volume reduction, these were applied to an HPTLC plate, which was developed in C/M/0.25M KCl, as described [7,8]. By using subunit B of cholera toxin linked to horseradish peroxidase (CtxB-HRP) to detect and quantify GM1 on the plate, we found that both the heart and colon contained significantly less GM1 than the controls (Figure 3). The same was true for GD1a, which was revealed after conversion to GM1 through the application of N’ase to the plate. Notably, it was not necessary to isolate ganglioside from the other lipids prior to thin layer analysis because most of these ran well ahead of GM1 on the plate and in any case did not react with CtxB-HRP.

**Figure 4:**
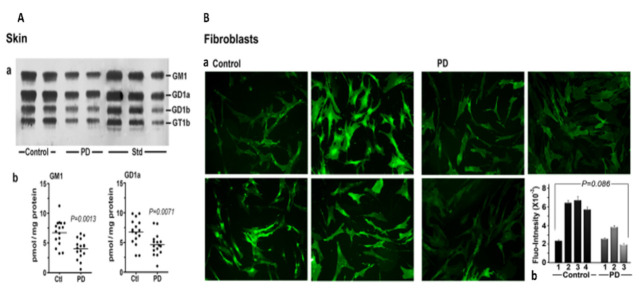
Gangliosides in skin and fibroblasts from PD patients and age-matched controls. (**A**): Skin (*n* = 16 in each group). Subpanel (**a**) is HPTLC, and subpanel (**b**) is densitometry quantification showing significant difference between PD and controls, calculated by Mann–Whitney rank sum U test. (**B**): (**a**) GM1-fluorescent images of cultured fibroblast cells from four non-PD controls and three age-matched PD patients were immunostained for GM1; (**b**) fluorescence intensity of GM1 staining in fibroblast cells, showing a marginal difference in GM1 by Student’s t-test (image used are adapted from [8] with permission granted by Glycoconjugate J., Copyright 2021 Springer).

We next considered skin, a tissue somewhat less well recognized as being involved in non-motor manifestations of PD (Figure 4). Seborrheic dermatitis is one such condition, causing lesions in the head and neck along with the upper trunk and sternum. GM1 analysis via HPTLC of skin samples revealed significant deficiency for the ganglioside as well as GD1a. An analysis of cultured fibroblasts using fluorescent immunohistochemistry showed a similar trend, although this did not quite reach significance—likely due to the limited number of samples available (Figure 4B). 

**Figure 5:**
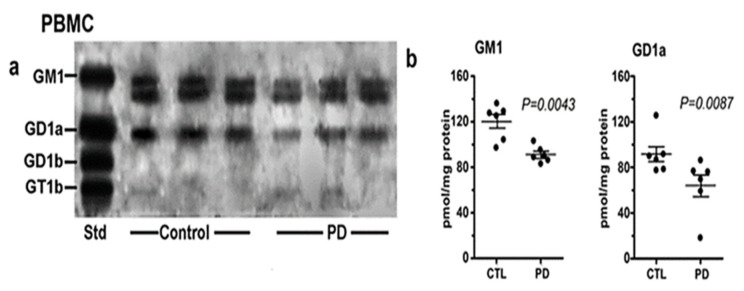
Gangliosides in PBMCs from PD patients and age-matched controls. These were analyzed with HPTLC (*n* = 6 in each group). Subpanel (**a**) is HPTLC, and subpanel (**b**) is densitometry quantification showing the statistical difference between PD and controls, calculated by Mann–Whitney rank sum U test (image used are adapted from [8] with permission granted by Glycoconjugate J., Copyright 2021 Springer).

Of considerable interest was our results with PBMC (Figure 5). These white blood cells showed a similar deficiency of both GM1 and GD1a as for PD neurons, despite their lack of direct neuronal involvement. We recently carried out a more detailed study of PBMC in which we found more severe GM1 deficiency in PD patients with glucocerebrosidase malfunction than in PD patients with more ordinary sporadic PD [9]. We accordingly proposed GM1 deficiency in PBMC as a method for early diagnosis of those two forms of PD. 

The impressive array of CNS and non-CNS symptoms revealed by Braak et al. [10,11] and others more recently [12,13] is consistent with the body-wide deficiency of GM1 we are proposing. In line with that, we have suggested that GM1 deficiency constitutes a major risk factor for sporadic PD, which pertains to 90% or more of PD cases with unknown etiology. Only 10% or so of PD cases are hereditary in nature, arising from specific genetic mutations [14].

## 4. Cause of GM1 Deficiency

It has become recognized that a major part of the GM1 deficiency is due to the aging process itself, consistent with the discovery of Svennerholm and coworkers that both GM1 and GD1a decrease progressively with age [15,16]. However, that alone would not explain the commonly found difference between PD subjects and age-matched controls, the latter also manifesting age-related decline without developing PD. This points to the existence of one or more additional factors that further depress GM1 and GD1a in PD. One such additional influence could be defective lysosomal hydrolase, as observed in our PBMC study [9]. That study involved glucocerebrosidase, which is the most prevalent of these, but it is noteworthy that potentially damaging variants of 50 or more less prevalent lysosomal storage disorder genes have been reported in PD cases [17]. Considerable attention has been given to aSyn, the degradation of which is mainly lysosomal, and the malfunction of that process can cause aSyn accumulation and aggregation. Significantly, GM1 was shown to promote autophagy-dependent removal of aSyn in a mouse model of PD [18]. 

Moreover, potential influence of the microbiome has to be considered, and it was recently studied in relation to both PD model and PD subjects [19].

A fairly recent study reported significantly reduced fecal short-chain fatty acids [20], which were shown to inhibit histone deacetylase [21,22]. Such inhibition promotes epigenetic activation of GM1 synthesis [23,24]. Environmental toxins are also potential inhibitors of GM1 synthesis [25].

Finally, the recent work of Niimi et al. points to enhanced degradation of GM1 as an alternative to impaired synthesis [26,27]. This was based on significant downregulation of glucosylceramide in PD. The higher activity of beta-galactosidase in the blood serum of PD patients was also observed. The proposal of enhanced degradation is worth further consideration.

## 5. GM1 Decreases in the Periphery as well as Brain

Concerning progressive decline of GM1—due to both age and additional factors—it is important to recognize that this occurs body wide and not only in the brain. Hence, this obviates the need to postulate prion-like movement of aggregated aSyn or to debate body first vs. brain first. Such prion-like movement undoubtedly does occur but likely on a limited scale. It is difficult to observe how this would account for PD manifestations in numerous and diverse locations such as gastrointestinal, cardiovascular, and dermatological neurons among others involved in PD. Since all neurons are dependent on an adequate level of GM1 for viability and neuronal functioning, those in the periphery would be expected to suffer dysfunction—with concomitant PD symptoms—more or less in parallel with those of CNS.

An important and frequently asked question concerns the underlying biochemistry that renders GM1 so essential to neuron function and long-term viability. In general, GM1 serves several essential functions through stereospecific association with particular proteins that preserves the stereospecificity necessary for the normal functioning of those proteins. Well-established examples include nerve growth factor (NGF) and brain-derived growth factor (BDNF), both of which have receptors tightly bound to GM1 for long term preservation of neuronal functioning (discussed below). We propose that aSyn constitutes an important addition to the list of such proteins since its binding of GM1 is what holds aSyn in alpha-helical, non-aggregating conformation [28,29]. The latter in vitro studies have been supplemented by in vivo demonstration in PD mouse models of GM1′s ability to disperse aSyn aggregates [6,30]. We further propose that this essential binding is carried out by the small pool of soluble GM1 [31,32], which was shown to bind soluble proteins (yet to be identified) [32]. Preliminary evidence for this was obtained in the aggregation of aSyn following depletion of cytosolic GM1 in cultured NG108-15 cells (supplement to Ledeen et al., 2021) [8]. A key point is that even a small deficiency of GM1 can disrupt the homeostatic balance, resulting in increasing aSyn aggregation as the deficiency continues.

## 6. GM1 and Neurotrophic Factors

As mentioned, the receptors for NGF (TrkA) and BDNF (TrkB) represent prime examples of proteins requiring association with GM1 to promote neuronal function and long-term neuronal viability in both the CNS and PNS [33,34]. We would add the receptor for glial cell line-derived neurotrophic factor (GDNF) to that, a nerve growth factor shown essential for the long-term viability of adult catecholaminergic neurons; importantly, this included DA neurons of the SNpc [35]. Our study, employing both PD tissues and a PD mouse model, revealed GM1 as an integral and essential component of the two-protein receptor complex [7]. More such receptors for additional factors may yet be discovered, but these three alone suggest that the wide array of neuronal types is dependent on receptor associated GM1. This also indicates the variety of CNS and PNS neurons destined to gradually become dormant and dysfunctional as GM1 continues to decline through aging in conjunction with various processes as suggested above.

It is worth mentioning that this does not exhaust the rather lengthy list of vital functions mediated by GM1 and its GD1a precursor. Many of these have been summarized in recent reviews [4,36].

## 7. Conclusions and Therapeutic Implications

We have endeavored to show systemic deficiency of GM1 ganglioside in all PD tissues examined to date, including non-neuronal cells (PBMC) as well as neurons of both the CNS and PNS. This has been accompanied by subnormal GD1a, the metabolic precursor of GM1. We have suggested possible causes of such deficiencies such as lysosomal dysfunction or microbiotic abnormality, which further reduce GM1 and GD1a due to natural aging. Some of the metabolic consequences of subnormal GM1 are outlined, including the misfolding and aggregation of aSyn as well as the failure of GDNF and other neurotrophic factors to fulfill their long-term viability and functional roles. Finally, it is important to mention a potential therapeutic solution for PD suggested by systemic GM1 deficiency—namely GM1 replacement therapy. This was first indicated in the application of GM1 to various PD animal models [37] but more forcefully in clinical trials conducted by Dr. Jay Schneider and coworkers. One of these involved a five-year open-label study in which GM1 was administered in two daily doses subcutaneously [38]. This resulted in improved UPDRS scores, and these motor scores show less disability after five years than at baseline. A randomized, controlled, delayed-start phase II trial was conducted next, which resulted in the slowing of symptoms over a two-year period [39]. It was speculated that the absence of more definitive disease-altering results was due to the limited ability of GM1 to enter the brain and neuron cytosol (e.g., to react with aSyn). It should be mentioned that the above clinical trials were conducted with GM1 from the bovine brain and such trials were terminated by FDA due to fear of prion protein contamination. However, it was shown that E. coli derived GM1 proved to be fully effective in a mouse PD model [40], but that too was discontinued due to financial difficulties of the sponsoring pharmaceutical company. However, the promising results obtained to date argue strongly in favor of continuing this line of therapeutic study. 

## Data Availability

Not applicable.
